# Modeling Ovo-vegetarian, Lacto-vegetarian, Pescatarian, and Vegan USDA Food Patterns and Assessing Nutrient Adequacy for Lactation among Adult Females

**DOI:** 10.1016/j.cdnut.2023.102034

**Published:** 2023-11-20

**Authors:** Julie M. Hess, Madeline E. Comeau, Kylie Swanson, Mandy Burbank

**Affiliations:** 1 US Department of Agriculture, Agriculture Research Services, Grand Forks Human Nutrition Research Center, Grand Forks, ND, United States; 2 University of North Dakota, Grand Forks, ND, United States; 3 Grand Forks Public Health, Grand Forks, ND, United States

**Keywords:** Dietary Guidelines for Americans, lactation, vegetarian, vegan, dietary patterns

## Abstract

**Background:**

Among its recommended dietary patterns for Americans, including lactating mothers, the 2020 Dietary Guidelines for Americans (DGA) includes a Healthy Vegetarian Dietary Pattern (HVDP). However, the DGA does not provide guidance for adapting the HVDP for vegetarians who avoid dairy (ovo-vegetarian) or eggs (lacto-vegetarian), eat fish (pescatarians), or avoid all animal foods (vegan).

**Objective:**

To determine whether models of the HVDP for different vegetarian diets could provide sufficient nutrition during lactation, a life stage with unique nutrient needs.

**Methods:**

Adaptations of the HVDP were developed at the 2200 and 2400 kcal levels using similar methods to the 2020 DGA. We compared these models with both the original HVDP and Dietary Reference Intakes (DRIs) for women ages 19 to 30 and ages 31 to 50 during lactation mo 1 to 12. All models were developed both with and without the addition of a multivitamin and -mineral prenatal supplement, commonly consumed by women throughout lactation.

**Results:**

All models (original HVDP, ovo-vegetarian, lacto-vegetarian, pescatarian, vegan) at all energy levels met the Adequate Macronutrient Distribution Ranges. Like the original HVDP and other dietary patterns in the DGA, the vegetarian adaptations in this study did not contain enough vitamin D, vitamin E, or choline to meet DRIs across all models and energy levels. With the prenatal supplement added, the models did not contain enough sodium, vitamin D, or choline. Some models also contained < 100% of the DRIs for sodium, zinc, vitamin A, and vitamin B6. Amounts of all other micronutrients met DRIs.

**Conclusions:**

Adaptations of the HVDP can provide adequate amounts of most nutrients, including nutrients of concern, during lactation to meet the needs of mothers during this life stage.

## Introduction

The 2020 Dietary Guidelines for Americans (DGA) was the first set of federal nutrition guidance in the United States to address nutrition needs during lactation. This DGA recommends 3 dietary patterns—a Healthy US-Style Dietary Pattern (HUSDP), a Healthy Mediterranean-Style Dietary Pattern, and a Healthy Vegetarian Dietary Pattern (HVDP). With the exceptions of vitamin D, vitamin E, and choline, the nutrients in these patterns are sufficient to meet the nutrient needs of most healthy Americans ages ≥2, including during lactation.

Lactation is a period of unique nutrition needs. Needs for folic acid and iron decrease during lactation, relative to pregnancy, though folate recommendations are still higher during lactation and pregnancy than during other life stages. Iodine and choline requirements also increase during lactation, as do protein and energy needs. Compared to prepregnancy energy requirements, an additional 330 kcal/d are needed during the first 6 mo of lactation, and an additional 400 kcal/d are needed during the second 6 mo of lactation [1]. Maternal diet continues to be important during breastfeeding both to ensure continued health of the mother as well as to support infant and early childhood health, as some of the amounts of micronutrients in human milk are correlated with maternal nutrition [1]. “Early childhood” throughout this manuscript refers to infancy through 2 y of age, as the American Academy of Pediatrics recommends continuing breastfeeding for 2 y or “as long as mutually desired by mother and child”[2]. The content of several vitamins (A, D, E, K, B_1_, B_2_, B_6_, B_12_, choline) and minerals (iodine, selenium) in human milk depends on maternal nutrient status and intake and “can be increased by maternal supplementation”[1].

Throughout this manuscript, we use the terms “woman” or “women” to describe people who lactate because most studies on nutrition and lactation to date have been conducted predominantly in populations “assigned female at birth” who also identify as “women.” We recognize that people of all genders can lactate [3]. In addition, although we use the term “breastfeeding” in this manuscript to reflect the language used in previous scientific work, “chestfeeding” is more inclusive terminology [3].

Women who are lactating tend to consume diets of higher quality relative to their nonpregnant and nonlactating peers but still tend to underconsume fruits, vegetables, dairy foods, and seafood [1]. Yet, there is relatively little data on dietary patterns during lactation, including the prevalence of vegetarian eating among women during this life stage. According to a 2019 survey conducted by the International Food Information Council [4], ∼ 23% of Americans self identify as vegetarian, pescatarian, vegan, or “vegetarian some days.” This survey also indicated that nearly half of Americans believe that plant alternatives are somewhat or much better for the environment. However, the top reason consumers report for trying plant-based alternatives to meat is curiosity about new foods, not environmental reasons [4]. While plant-based eating and plant-based foods are trends among US consumers [5], there has been little formal research on the adoption of actual vegetarian and vegan diets. Because not much research has been conducted on this topic overall, there is even less data available on plant-based eating and the nutrient adequacy of plant-based eating patterns during lactation specifically.

The DGA recommends that women following vegetarian or vegan diets during lactation remain especially attentive to their intakes of iron and vitamin B_12_ as well as choline, zinc, iodine, eicosopentanoic acid (EPA), and docosahexaenoic acid (DHA) and to consult with their healthcare providers regarding supplements [1].

Beyond the HVDP, which contains dairy foods and eggs (a lacto-ovo-vegetarian diet), the 2020 DGA does not provide guidance for vegetarian diets during lactation. That is, the 2020 DGA does not provide guidance for vegetarians who avoid dairy (ovo-vegetarian), avoid eggs (lacto-vegetarian), eat fish (pescatarians), or avoid all animal foods (vegan). The purpose of this study, therefore, was to assess nutrient adequacy of different adaptations of the HVDP (e.g., ovo-vegetarian, lacto-vegetarian, pescatarian, and vegans). For each of these adaptations, we used procedures similar to the DGA for food pattern modeling.

## Methods

The HVDP was adapted at the 2400 and 2200 kcal levels, as patterns at these energy levels are specified in the DGA as those most likely to align with energy needs for women 19 to 30 y and 31 to 50 y, respectively, during lactation mo 1 to 12. Specific adaptations to the HVDP made for each of the specific iterations of vegetarian diets are detailed below and in Table 1. Ovo-vegetarian, lacto-vegetarian, pescatarian, and vegan adaptations of the HVDP were developed at the 2200 and 2400 kcal levels, and all models were developed both with and without the addition of a prenatal supplement. Because this research was conducted using food pattern modeling of data already in the public domain, Institutional Review Board approval was not required for this study. The 2020 Dietary Guidelines for Americans Scientific Advisory Committee (DGAC) Food Pattern Modeling Report [6] was used to generate an Excel document of the food groups and nutrients in the HVDP and make adjustments for each of the dietary patterns modeled in this study. The Food Pattern Modeling Report utilizes data from the 2015-2016 National Health and Nutrition Examination Survey (NHANES). NHANES survey protocol approvals are conducted by the Research Ethics Review Board at the Centers for Disease Control and Prevention, National Center for Health Statistics [7].

**TABLE 1:** Daily or Weekly Amounts from Food Groups, Subgroups, and Components in the 2200 kcal/d Healthy Vegetarian Dietary Pattern (HVDP) and Modeled Variations^1^

Food Groups	HVDP	Model 1: Ovo-Vegetarian	Model 2: Vegan	Model 3: Lacto-Vegetarian	Model: 4 Pescatarian
Vegetables, cup eq/d	3	3	3	3	3
Dark green vegetables, cup eq/wk	2	2	2	2	2
Red and orange, cup eq/wk	6	6	6	6	6
Beans, peas, lentils, cup eq/wk	2	2	2	2	2
Starchy vegetables, cup eq/wk	6	6	6	6	6
Other vegetables, cup eq/wk	5	5	5	5	5
Fruits, cup eq/d	2	2	2	2	2
Grains, oz-eq/d	7.5	7.5	7.5	7.5	7
Whole grains	4	4	4	4	4
Refined grains	3.5	3.5	3.5	3.5	3
Dairy, cup eq/d	3	0	0	3	3
DairyALT, cup eq/d	0	3	3	0	0
Protein foods, oz-eq/d	3.5	3.5	3.5	3.5	3.5
Eggs, oz-eq/wk	3	3	0	0	3
Beans, peas, lentils, oz-eq/wk	6	6	7	7	6
Soy products, oz-eq/wk	8	8	9	9	8
Seafood, oz-eq/wk	0	0	0	0	9
Nuts, seeds, oz-eq/wk	7	7	8	7	6
Oils, g/d	29	29	29	29	29
Discretionary calories, kcal/d	290	290	290	290	290

1 One cup eq = 2.37 dL. 1 oz-eq = 28.35 g. Cup eq, cup equivalent; DairyALT, dairy alternative; HVDP, Healthy Vegetarian Dietary Pattern; oz-eq, ounce equivalent.

## Ovo-vegetarian (Model 1)

Ovo-vegetarian diets include eggs but exclude dairy foods. Therefore, we replaced each cup equivalent (cup eq) of dairy foods in the HVDP with a food group comprised of the only nondairy alternatives recognized in the 2020 DGA as part of the “dairy food group,” namely, fortified soy yogurt and soy milk. The nutrient and food makeup of this food group, which we describe as the dairy alternative (ALT) group, has been published [5].

## Vegan (Model 2)

For the vegan model, which excludes both dairy foods and eggs, we replaced dairy with the dairyALT group and eggs with equivalent amounts of plant-based protein foods (i.e., beans, peas, and lentils; nuts and seeds; soy foods).

## Lacto-vegetarian (Model 3)

Lacto-vegetarian diets include dairy foods but exclude eggs. To replace eggs in the HVDP, which are considered a protein food, we increased the servings of the other protein foods by equivalent amounts.

## Pescatarian (Model 4)

For our pescatarian dietary pattern, we added the recommended amounts of seafood from the HUSDP in the 2020 DGA to the HVDP. For diets during lactation (2200 and 2400 kcal) for adult women, these amounts are 9 ounce-equivalents (oz-eq)/wk and 10 oz-eq/wk, respectively. For the 2200 kcal pescatarian diet, the addition of 9 oz-eq/wk of seafood would add ∼ 50 kcal/d to the diet. To mitigate the change in energy, we decreased the servings of refined grains by 0.5 oz-eq/d (from 3.5 oz-eq/d to 3 oz-eq/d), a change of ∼ 43 kcal.

## Prenatal Supplements

Each model was created both with and without the addition of a prenatal supplement since most lactating women take a multivitamin and -mineral (MVM) prenatal supplement [8]. However, because there is no standard nutrient content of prenatal supplements, we developed an “average” prenatal supplement using data from the Office of Dietary Supplements (ODS) Dietary Supplement Label Database (DSLD) [9]. In this database, we conducted a search for MVM products labeled for use during pregnancy and lactation, which yielded 157 products. For each nutrient with a value > 0 as the mode, we calculated the average amount of that micronutrient. Our sample prenatal supplement included calcium, folic acid, iron, niacin, riboflavin, vitamin A, vitamin B_6_, vitamin C, and vitamin E (Table 2). With nutrients that had multiple names, for example, riboflavin and “vitamin B_2,_” or for nutrients that had several forms, such as calcium (calcium ascorbate, calcium carbonate, calcium citrate, calcium formate, calcium malate), the additional names or forms of the nutrient were added to the total, and thus average, of that nutrient.

**TABLE 2:** Average Prenatal Supplement Developed from the Dietary Supplement Label Database

Common nutrients in prenatal supplements	Average amount of nutrient in prenatal supplements
Calcium (mg)	211
Folate/Folic acid (μg DFE)	1155
Iron (mg)	27
Niacin (mg)	18
Riboflavin (mg)	3.1
Vitamin A (μg RAE)	1008
Vitamin B_6_ (mg)	7.8
Vitamin C (mg)	108
Vitamin E (mg)	14.6
Zinc (mg)	18.5
Vitamin B_12_ (μg)	18.9

## Women’s MVM Supplement

Prenatal supplements are recommended to support the nutrition of pregnant individuals [10], but these supplements are not intended for use during lactation. Using methods like those used for generating an “average” prenatal supplement, we also developed an “average” women’s MVM supplement based on products listed in ODS’s DSLD. The search strategy included the terms “women’s multivitamin and mineral” with filters: “on market,” “Multi-Vitamin and Mineral (MVM),” “All Adults and Children 4 Years and Above,” and under “label statements and claims:” “women.” This search yielded 194 unique labels. From our search results, we removed MVM supplements that were intended for those “pregnant and lactating” and for “children 4 and under.” We additionally removed any MVM that contained the keywords “prenatal,” “prenate,” “PNV,” “senior,” “50+,” “fifty plus,” “40+,” “over 50,” “65+,” “kids,” “children,” or “teen,” as well as 2 duplicate results to reflect MVMs that are intended for women of reproductive age. Our final list yielded 156 unique supplements. Each nutrient with a value greater > 0 for the mode was averaged, and that amount was included in our final list of nutrients in the women’s MVM composite. Our sample included vitamin B_12_, vitamin B_6_, vitamin C, and calcium.

## Results

### Without prenatal supplements

All models (original HVDP, ovo-vegetarian, lacto-vegetarian, pescatarian, vegan) at all energy levels provided adequate energy and macronutrients to meet the Acceptable Macronutrient Distribution Ranges (AMDRs). The original HVDP model provided < 50% of the DRI for vitamin D only, whereas nutrients between 50 and 100% of the DRIs included vitamin A, vitamin E, choline, and sodium (at the 2400 kcal level).

Like the original HVDP and other dietary patterns in the DGA, the vegetarian adaptations in this study did not contain enough vitamin D, vitamin E, or choline to meet DRIs across all models and energy levels. In addition to these nutrients, some models also contained less than 100% of the DRIs for sodium, zinc, vitamin A, and vitamin B_6_. These additional nutrient shortfalls are detailed below. Amounts of all other micronutrients available in these models, including calcium, folic acid, and iron, were provided in adequate amounts to meet the DRIs (TABLE 3, TABLE 4, TABLE 5, TABLE 6).

**TABLE 3:** Macronutrients and Micronutrients in the 2200 kcal/d Healthy Vegetarian Dietary Pattern (HVDP) and Ovo-Vegetarian and Vegan Models for Lactation

	Dietary Reference Intakes (Females 31–50 y; Lactation Mo 1–12)	Healthy Vegetarian Dietary Pattern (HVDP)	Model 1: Ovo- vegetarian pattern	% of DRI	Model 2: Vegan at 2200 kcal	% of DRI
MACRONUTRIENTS						
Calories (kcal)	2200	2200	2275	103.41	2271	103.23
Protein (g)	71	85	77	108.23	77	108.24
Carbohydrate (g)	210	277	281	133.80	28	134.74
Fiber (g)	31	33.8	35	112.05	36	114.53
Total Fat (g)	20–35% kcals	58.5	67	Adequate	65	Adequate
Saturated Fat (g)	<10%	11.0	11	Meets criteria	10	Meets criteria
Monounsaturated Fatty Acids (g)	n/a	21.0	23	n/a	22	n/a
Polyunsaturated Fatty Acids (g)	n/a	22.3	28	n/a	28	n/a
18:2 Linoleic acid (g)	13	19.7	23	180.31	24	180.94
18:3 Linolenic acid (g)	1.3	2.54	3	232.40	3	234.38
EPA (20:5 n-3) (g)	n/a	0.000	0.000	n/a	0.000	n/a
DHA (22:6 n-3) (g)	n/a	0.009	0.009	n/a	0.000	n/a
Cholesterol (mg)	As low as possible	106	83	n/a	3	n/a
**MINERALS**						
Calcium (mg)	1000	1392	1375	137.54	1377	137.73
Iron (mg)	9	18	21	233.44	21	236.38
Magnesium (mg)	320	419	445	139.20%	452	141.38
Phosphorus (mg)	700	1718	1345	192.12	1344	191.93
Potassium (mg)	2800	3575	3626	129.50	3644	130.14
Sodium (mg)	2300	1573	1317	57.24	1310	56.97
Zinc (mg)	12	12	10	87.22	10	87.22
Copper (mg)	1.3	2	3	211.12	3	215.89
Selenium (μg)	70	87	81	115.71	75	107.08
**VITAMINS**						
Vitamin A, RAE (μg)	1300	916	977	75.14	945	72.68
Vitamin E, AT (mg)	19	11.11	12	61.81	12	62.18
Vitamin D (IU)	600	223	373	62.16	355	59.12
Vitamin C (mg)	120	142	149	124.56	150	124.61
Thiamin (mg)	1.4	2.01	2	141.60	2	142.27
Riboflavin (mg)	1.6	1.93	2	139.13	2	133.04
Niacin (mg)	17	18.6	20	119.10	20	120.45
Vitamin B_6_ (mg)	2	2.03	2	98.41	2	98.27
Vitamin B_12_ (μg)	2.8	3.98	7	246.99	7	238.39
Choline (mg)	550	318	399	72.60	343	62.42
Vitamin K (μg)	90	170	189	210.51	190	210.84
Folate, DFE (μg)	500	697	729	145.80	602	120.40

**TABLE 4:** Macronutrients and micronutrients in the 2200 kcal/d lacto-vegetarian and pescatarian models for lactation

	Dietary Reference Intakes (Females 31–50 y; Lactation Mo 1–12)	Model 3: Lacto-Vegetarian (2200 kcal)	% of DRI	Model 4: Pescatarian (2200 kcal)	% of DRI
ENERGY/MACRONUTRIENTS					
Calories (kcal)	2200	2197	99.87	2209	100.43
Protein (g)	71	85	119.85	92	129.68
Carbohydrate (g)	210	279	132.98	269	128.25
Fiber (g)	31	35	111.66	33	108.02
Total fat (g)	20–35% kcals	58	Adequate	60	Adequate
Saturated fat (g)	<10% kcals	10	Meets criteria	11	Meets criteria
Monounsaturated fatty acids (g)	n/a	21	n/a	21	n/a
Polyunsaturated fatty acids (g)	n/a	22	n/a	23	n/a
18:2 Linoleic acid (g)	13	20	151.86	20	150.86
18:3 Linolenic acid (g)	1.3	3	197.46	3	195.94
EPA (20:5 n-3) (g)	n/a	0.000	n/a	0.082	n/a
DHA (22:6 n-3) (g)	n/a	0.000	n/a	0.185	n/a
Cholesterol (mg)	As low as possible	25	n/a	135	n/a
**MINERALS**					
Calcium (mg)	1000	1393	139.35	1392	139.19
Iron (mg)	9	19	207.71	18	202.48
Magnesium (mg)	320	426	133.15	427	133.42
Phosphorus (mg)	700	1716	245.16	1794	256.26
Potassium (mg)	2800	3593	128.32	3676	131.30
Sodium (mg)	2300	1567	68.13	1617	70.31
Zinc (mg)	12	12	103.47	13	106.09
Copper (mg)	1.3	2	142.05	2	140.44
Selenium (μg)	70	81	115.27	101	144.95
VITAMINS					
Vitamin A, RAE (μg)	1300	884	68.01	926	71.20
Vitamin E, AT (mg)	19	11	58.86	11	60.07
Vitamin D (IU)	600	205	34.12	312	51.99
Vitamin C (mg)	120	142	118.28	142	118.45
Thiamin (mg)	1.4	2	144.41	2	140.97
Riboflavin (mg)	1.6	2	114.29	2	119.99
Niacin (mg)	17	19	110.57	20	117.37
Vitamin B_6_ (mg)	2	2	101.27	2	106.30
Vitamin B_12_ (μg)	2.8	4	133.39	5	189.27
Choline (mg)	550	262	47.57	341	62.02
Vitamin K (μg)	90	170	189.27	170	188.76
Folate, DFE (μg)	500	571	114.20	681	136.20

**TABLE 5:** Macronutrients and micronutrients in the 2400 kcal/d Healthy Vegetarian Dietary Pattern (HVDP) and ovo-vegetarian and vegan models for lactation

	Dietary Reference Intakes (Females 19–30 y; Lactation Mo 1–12)	Healthy Vegetarian Dietary Pattern (HVDP)	Model 1: Ovo-Vegetarian (2400 kcal)	% of DRI	Model 2: Vegan (2400 kcal)	% of DRI
ENERGY/MACRONUTRIENTS						
Calories (kcal)	2400	2404	2478	103.26	2474	103.10
Protein (g)	71	91	83	116.87	83	116.93
Carbohydrate (g)	210	297	301	143.32	303	144.28
Fiber (g)	31	36.6	37	120.82	38	123.34
Total fat (g)	20–35% kcals	63.2	71	Adequate	70	Adequate
Saturated fat (g)	<10% kcals	11.7	12	Meets criteria	11	Meets criteria
Monounsaturated fatty acids (g)	n/a	22.7	24	n/a	24	n/a
Polyunsaturated fatty acids (g)	n/a	24.2	30	n/a	30	n/a
18:2 Linoleic acid (g)	13	21.3	25	193.25	25	194.02
18:3 Linolenic acid (g)	1.3	2.74	3	247.48	3	249.54
EPA (20:5 n-3) (g)	n/a	0.00	0	n/a	0	n/a
DHA (22:6 n-3) (g)	n/a	0.01	0	n/a	0	n/a
Cholesterol (mg)	As low as possible	106	84	n/a	4	n/a
**MINERALS**						
Calcium (mg)	1000	1436	1420	141.98	1422	142.16
Iron (mg)	9	20	23	254.76	23	257.53
Magnesium (mg)	320	450	476	153.50	483	155.83
Phosphorus (mg)	700	1822	1450	207.10	1447	206.78
Potassium (mg)	2800	3704	3755	134.11	3774	134.77
Sodium (mg)	2300	1684	1427	62.05	1420	61.73
Zinc (mg)	12	13	11	94.89	11	94.88
Copper (mg)	1.3	2	3	222.10	3	226.81
Selenium (μg)	70	94	88	126.20	82	117.58
VITAMINS						
Vitamin A, RAE (μg)	1300	935	996	76.63	964	74.17
Vitamin E, AT (mg)	19	11.94	13	66.17	13	66.63
Vitamin D (IU)	600	227	377	62.76	358	59.73
Vitamin C (mg)	120	143	150	125.12	150	125.17
Thiamin (mg)	1.4	2.18	2	153.92	2	154.59
Riboflavin (mg)	1.6	2	2	143.98	2	137.91
Niacin (mg)	17	20.2	22	128.81	22	130.22
Vitamin B_6_ (mg)	2	2.13	2	103.50	2	103.38
Vitamin B_12_ (μg)	2.8	4.10	7	251.46	7	242.86
Choline (mg)	550	332	413	75.18	357	64.96
Vitamin K (μg)	90	174	193	214.91	194	215.24
Folate, DFE (μg)	500	626	657	131.40	654	130.80

**TABLE 6:** Macronutrients and micronutrients in the 2400 kcal/d lacto-vegetarian and pescatarian models for lactation

	Dietary Reference Intakes (Females 19–30 y; Lactation Mo 1–12)	Model 3: Lacto-vegetarian (2400 kcal)	% of DRI	Model 4: Pescatarian (2400 kcal)	% of DRI
ENERGY/MACRONUTRIENTS					
Calories (kcal)	2400	2401	100.02	2478	103.26
Protein (g)	71	91	128.37	83	116.87
Carbohydrate (g)	210	299	142.52	301	143.32
Fiber (g)	31	37	120.47	37	120.82
Total Fat (g)	20–35% kcals	62	Adequate	71	Adequate
Saturated Fat (g)	<10% kcals	11	Meets criteria	12	Meets criteria
Monounsaturated Fatty Acids (g)	n/a	22	n/a	24	n/a
Polyunsaturated Fatty Acids (g)	n/a	24	n/a	30	n/a
18:2 Linoleic acid (g)	13	21	164.94	25	193.25
18:3 Linolenic acid (g)	1.3	3	212.62	3	247.48
EPA (20:5 n-3) (g)	n/a	0	n/a	0	n/a
DHA (22:6 n-3) (g)	n/a	0	n/a	0	n/a
Cholesterol (mg)	As low as possible	26	n/a	84	n/a
MINERALS					
Calcium (mg)	1000	1438	143.78	1420	141.98
Iron (mg)	9	21	228.87	23	254.76
Magnesium (mg)	320	457	147.33	476	153.50
Phosphorus (mg)	700	1820	260.00	1450	207.10
Potassium (mg)	2800	3722	132.95	3755	134.11
Sodium (mg)	2300	1676	72.88	1427	62.05
Zinc (mg)	12	13	111.13	11	94.89
Copper (mg)	1.3	2	152.96	3	222.10
Selenium (μg)	70	88	125.77	88	126.20
VITAMINS					
Vitamin A, RAE (μg)	1300	903	69.50	996	76.63
Vitamin E, AT (mg)	19	12	63.32	13	66.17
Vitamin D (IU)	600	208	34.73	377	62.76
Vitamin C (mg)	120	143	118.85	150	125.12
Thiamin (mg)	1.4	2	156.74	2	153.92
Riboflavin (mg)	1.6	2	119.16	2	143.98
Niacin (mg)	17	20	120.34	22	128.81
Vitamin B_6_ (mg)	2	2	106.38	2	103.50
Vitamin B_12_ (μg)	2.8	4	137.86	7	251.46
Choline (mg)	550	276	50.12	413	75.18
Vitamin K (μg)	90	174	193.68	193	214.91
Folate, DFE (μg)	500	623	124.60	611	122.20

#### Model 1—Ovo-Vegetarian

Nutrients < 100% of DRI for lactating females 31 to 50 y during lactation mo 1 to 12 at the 2200 kcal level included sodium (57.24% of the DRI), zinc (87.22%), vitamin A (75.14%), and vitamin B_6_ (98.41%). At the 2400 kcal level for lactating females 19 to 30 y during mo 1 to 12, nutrients provided at < 100% of the DRI in the ovo-vegetarian model also included sodium (62.05%), zinc (94.89%), and vitamin A (76.63%).

#### Model 2—Vegan

No micronutrients were provided in amounts < 50% of the DRIs set for lactating females 31 to 50 y during lactation mo 1 to 12 (2200 kcal); however, levels of sodium (56.97%), zinc (87.22%), and vitamin A (72.68%) were provided in amounts < 100% of the DRI. For lactating females 19 to 30 y consuming 2400 kcal, nutrients at levels < 100% of the DRI also included sodium (61.73%), zinc (94.88%), and vitamin A (74.17%).

#### Model 3—Lacto-vegetarian

Sodium (72.88%; 68.13%) and vitamin A (69.5%; 68.01%) were the only 2 additional nutrients provided in amounts < 100% of the DRI in the lacto-vegetarian patterns for lactating females 19 to 30 and 31 to 50 y, respectively.

#### Model 4—Pescatarian

For lactating females 31 to 50 y of age on a pescatarian diet, sodium (70.31%) and vitamin A (71.20%) were below the DRI recommendations, whereas for lactating females 19 to 30 y, zinc was also below the DRI recommendations (94.89% of DRI).

### With prenatal supplements

With the addition of prenatal supplements, the only nutrient < 50% of the DRI in the original HVDP at both energy levels was vitamin D, though choline and sodium were also < DRI recommendations (∼ 70% and 60% of the DRIs, respectively). For all vegetarian models, vitamin D, choline, and sodium were the only micronutrients < the DRIs.

Because prenatal supplements are intended for use during pregnancy, adding these MVMs into our models for lactation increased the amounts of some micronutrients to a level that exceeded the Tolerable Upper Intake Levels (UL), including folic acid and iron. These nutrients and ULs are addressed in the Discussion section.

#### *Model 1*—*Ovo-Vegetarian*

No nutrients were provided < 50% of the DRIs in these models. Sodium, vitamin D, and choline were between 25 and 40% below DRI recommendations.

#### *Model 2*—*Vegan*

Similar to the ovo-vegetarian models, no nutrients were provided in amounts < 50% of the DRIs for lactating females in the vegan models. Sodium, vitamin D, and choline were ∼ 35 to 45% below recommendations.

#### *Model 3*—*Lacto-Vegetarian*

The lacto-vegetarian models were the only models where vitamin D amounts fell < 50% of the DRI (34% of the DRI in the 2200 kcal model and 35% of the DRI in the 2400 kcal model). Choline was also provided in amounts < 50% of the DRI in the 2200 kcal model (48%) but was > 50% in the 2400 kcal model. Sodium was provided at ∼ 70% of the adequate intake level at both energy levels.

#### *Model 4*—*Pescatarian*

Like the ovo-vegetarian and vegan models, no nutrients were provided in amounts < 50% of the DRIs in the pescatarian models. Sodium, vitamin D, and choline were provided in amounts between 50 and 100% of the DRIs.

### Women’s MVM Supplement

Our composite women’s MVM supplement included 4 micronutrients: calcium (277.09 mg) and 3 vitamins: vitamin B_12_ (26.64 μg), vitamin B_6_ (3.73 mg), and vitamin C (92.1 mg) (Supplemental Table 1).

## Discussion

The vegetarian adaptations of the HVDP for lacto-vegetarian, ovo-vegetarian, vegan, and pescatarian diets provided adequate amounts of all macronutrients and most micronutrients, including nutrients of concern, during lactation. The 2020 DGAC identifies food components of public health concern for underconsumption among women who are lactating, including vitamin D, calcium, dietary fiber, and potassium [11]. The DGAC also notes that lactating women may also have intakes below the Estimated Average Requirement (EAR) for folate, magnesium, copper, thiamin, vitamin A, and zinc [11]. Dietary supplements contribute to the intake of some important nutrients during lactation; however, continuing to take prenatal supplements during lactation also means some women will exceed recommendations for folic acid and iron [11].

Folic acid is listed as a nutrient at risk for overconsumption among women who are lactating [11]. From the pregnancy DRI of 600 μg/d, the DRI drops to 500 μg/d during lactation. Our prenatal supplement composite contained 1155 μg of folic acid, which exceeds the 1000 μg/d UL for folate/folic acid [12]. NHANES data from 1999 to 2014 indicate that 70% of women take supplements during lactation, with about half of these women continuing to take prenatal supplements during lactation [11]. The modeled dietary patterns in this manuscript, with the addition of a prenatal supplement, exceed the folic acid UL of 1000 μg/d [12]. However, these patterns are models, and it is unknown how many women may actually be at risk for exceeding the UL for folic acid consumption. NHANES data from 2007 to 2010 indicate that < 3% of women ages 14 to 50 y obtain > 1000 μg/d of folic acid from the combination of food, beverages, and dietary supplements [13]. In addition, high intakes of folic acid present little known risk of toxicity. The UL established for folate applies only to supplements and fortified foods; food sources have not been reported to cause adverse effects [12]. An indirect effect of excessive folic acid consumption, however, is masking underlying pernicious anemia, an anemia characterized by a vitamin B_12_ deficiency that can lead to adverse neurologic effects [14]. Since vegetarian diets, specifically those that contain little to no animal products, provide negligible amounts of vitamin B_12_, vegetarians and infants/young children of vegan women are at risk of deficiency [15]. Among the other micronutrients discussed in this paper, vitamin B_12_ supplementation is recommended by the Academy of Nutrition and Dietetics [16], the 2020 DGA [17], the American College of Obstetrics and Gynecologists [10], and the National Institutes of Health Office of Dietary Supplements [15] for vegetarians across all age groups to consider. Vitamin B_12_ was provided by both the composite prenatal supplement developed from DSLD data as well as from the average women’s MVM developed from DSLD data.

Similarly, iron also may pose a risk for overconsumption during lactation due to supplementation. The recommendation for iron during lactation is 9 mg/d for females 19 to 50 y, which is < 27 mg/d recommended throughout pregnancy. Reasons behind the lower recommended amount, compared with pregnancy, are due to assumed lactational amenorrhea in which menstruation is not occurring for the first 6 mo postpartum and, therefore, monthly blood loss depleting iron stores is not occurring during this time. Yet, since many women continue to take prenatal supplements throughout lactation, and many of these supplements contain iron, lactating women may be exceeding their iron needs. The UL for iron set for pregnancy and lactation is 45 mg/d [18]. Risks of iron overconsumption are generally associated with dietary supplement intake (versus intake of iron from foods) and can cause gastrointestinal distress and reduce zinc absorption [18]. However, if a parent chooses not to breastfeed, menstruation may return around 6 wk postpartum, and thus, iron needs increase back to the 18 mg/d DRI for menstruating adults 19 to 50 y [19]. The need for iron supplementation should be evaluated by a qualified healthcare provider on an individual basis.

In addition, postpartum blood loss may lead to a significant loss of iron, which may lead to postpartum anemia in which iron stores must be replaced to prevent complications for both mother and infant. The FAO and WHO assume blood losses during birth include the loss of ∼ 250 mg of iron (21). European organizations, including The German Nutrition Society (DGE), recommend an intake of 20 mg/d of iron postnatally, regardless of breastfeeding status [20]. Given the broad potential range of iron needs postpartum, women should discuss appropriate postnatal iron intake and/or continuation of prenatal supplements during lactation with their healthcare provider.

Although our models contained sufficient amounts of dietary iron to meet the DRIs during lactation, it is important to specify the differences between heme (animal sources) and nonheme iron (plant sources) and their different absorption rates [1]. Bioavailability of iron from mixed diets is ∼ 14 to 18% but is only 5 to 12% from vegetarian diets; therefore, the Recommended Dietary Allowance (RDA) for vegetarians is 1.8-fold higher than for people who eat meat, poultry, and seafood [18]. The 2020-2025 DGA does not account for the increased iron RDA and the differences in absorption with its food group recommendations in the HVDP for age, sex, and pregnancy/lactation status [1]. Without these data, we are unable to assess the impact of bioavailability differences in our models of vegetarian diets.

In addition to iron, the 2020 DGAC also specifically encourages consumption of foods rich in “choline, magnesium, protein, fiber, and vitamins A, D, and E” among women who are lactating [1]. According to the 2020 DGAC Report, “approximately 16 percent of women who are lactating consume less than recommended amounts of protein foods”[11]. We do not have data on the typical eating patterns and dietary choice of vegetarians, whether lactating or not. In the absence of this information, the DGA recommends that “women following a vegetarian or vegan diet during pregnancy or lactation may need to take special care to ensure nutrient adequacy”[1].

Currently, FoodDataCentral does not provide data regarding amounts of some critical nutrients during lactation, including iodine [21]. Therefore, we do not have complete information on whether the diets modeled either in the DGA or in this study provide adequate amounts of all essential nutrients during lactation. Similarly, although DHA/EPA are cited in the 2020 DGAC as important during lactation to support neural development of the child, and their concentration in human milk depends on the maternal diet, we are unable to assess the “adequacy” of these nutrients in our models as a DRI has not yet been established during lactation for these nutrients [21]. Our pescatarian models contained 9 to 10 oz-eq/wk of seafood equivalents and, therefore, ∼ 0.08 g/d of EPA and 0.2 g/d of DHA in the 2200 kcal/d model and 0.09 g/d of EPA and 0.2 g/d DHA in the 2400 kcal/d model. However, there is currently no evidence to “determine the relationship between maternal seafood intake during lactation and neurocognitive development in the child”[11]. Nonetheless, the 2020 DGAC encourages lactating females to consume a variety of seafood low in mercury and high in omega-3 fatty acids [11].

Another option to consider in maintaining nutrient adequacy during lactation includes the use of a women’s MVM in lieu of a prenatal supplement. The Centers for Disease Control and Prevention (CDC) point out that an MVM supplement may be necessary during lactation, especially if the individual is following a vegetarian or vegan diet [22]. This recommendation comes from increasing needs for choline and iodine during lactation, and diet alone may not be adequate to meet the unique nutritional needs to support the mother and growing child. Neither the “average” prenatal supplement nor the women’s MVM supplement from DSLD data indicate that iodine and choline are generally provided in these products. Therefore, the CDC's suggestion that a multivitamin “may be necessary” under advisement from a healthcare provider is especially important [22].

Interestingly, our women’s MVM composite provided no iron or folic acid rather than providing *less* iron and folic acid than our prenatal MVM. Additionally, the women’s MVM supplement did not provide listed amounts of nutrients cited for concern during lactation, including iodine, choline, zinc, or EPA/DHA (1). Instead, our composite contained 3 vitamins (C, B_12_, B_6_) and 1 mineral (calcium). From this analysis and in support of the current literature, discussing MVM supplementation during lactation with a healthcare provider is important to ensure that women have adequate nutrient intake during this stage.

Vitamin B_12_ was also provided in amounts far exceeding the DRI in our modeled diets with prenatal supplements added (>800% of the DRI). Although B_12_ is a nutrient of concern for those following vegetarian or vegan dietary patterns, especially during lactation, all 8 models provided adequate amounts of B_12_ before the addition of a supplement. Our models that used the dairyALT composite in lieu of dairy foods (ovo-vegetarian, vegan) provided more vitamin B_12_ at both the 2200 and 2400 kcal levels than the dairy-containing models (lacto-vegetarian, pescatarian). The dairyALT group provides 5.70 μg B_12_ in 3 cup eq compared with 2.76 μg from 3 cup eq of the dairy group. Therefore, a vitamin B_12_-containing supplement may not be necessary if adequate intake of vitamin B_12_-containing foods like fortified soy yogurt and soy milk are consumed.

Finally, whereas we are able to model adequacy of these dietary pattern adaptations for females who are lactating, DRIs have not yet been established for the postpartum period for people who are not breast/chestfeeding. Not all parents choose to or have the ability to feed their young child human milk. Therefore, there is currently a gap in recommendations since there are currently no nutrition recommendations for birthing parents postpartum who do not breast/chest feed.

## Limitations

While these models provided estimates for macronutrient and micronutrient adequacy of various vegetarian dietary patterns, this study has limitations. Food pattern models are based upon servings provided in the DGA and do not represent individual dietary habits and patterns necessarily reflective of eating patterns. Modeling studies such as this can provide context and feasibility prior to conducting future research or updating dietary guidance but cannot offer specific dietary guidance as a conclusion of the study. Another limitation of this study includes the lack of ability to assess iodine status. Iodine is a commonly cited nutrient of concern for those who follow vegetarian dietary patterns, and additionally, it becomes of particular importance during lactation to support infant and early childhood development. The DRI increases from 220 μg/d during pregnancy to 290 μg/d during lactation to support nutrient needs of both parent and infant [23]. Currently, the Food Pattern Modeling Report does not provide iodine amounts for food items; the development of this database is currently in progress [24]. Lastly, we were unable to assess individual nutrient bioavailability that may differ between animal and plant sources of various foods and nutrients provided by the models. Although we acknowledge these differences do exist, the Food Pattern Modeling Report does not provide this information at this time.

## Conclusion

The dietary patterns modeled in this study meet DRI recommendations for most nutrients, yet these patterns do not represent the actual dietary intake of vegetarian women during lactation but rather idealized diets for lactation. The results of this study indicate that vegetarian and vegan diets can provide adequate macronutrients and micronutrients if DGA recommendations are followed closely. Yet there are few data on the prevalence of vegetarian dietary habits among the general adult population as well as among smaller populations such as lactating women. To create more realistic patterns and to further explore the nutrient adequacy of vegetarian and vegan patterns during lactation, more cross-sectional research is needed on typical vegetarian eating habits during lactation.

## Author contributions

JMH and MEC designed the study. MEC and KS collected data and conducted data analysis. JMH, MEC, and KS wrote the first draft. MB reviewed and edited the manuscript. All authors have read and approved the final manuscript.

## Funding

Supported by USDA Agricultural Research Service project grant #3062-51000-057-00D

## Data availability

Data described in the manuscript is publicly and freely available without restriction as all data Data Availability Data described in the manuscript is publicly and freely available without restriction as all data used in this manuscript is in the public domain used in this manuscript is in the public domain.

## Conflict of interest

The authors report no conflicts of interest.
