# Supplementary material for: Modeling Ovo-vegetarian, Lacto-vegetarian, Pescatarian, and Vegan USDA Food Patterns and Assessing Nutrient Adequacy for Lactation among Adult Females
Source: Curr Dev Nutr. 2023 Nov 20;7(12):102034. doi: 10.1016/j.cdnut.2023.102034 (PMC10733672; doi:10.1016/j.cdnut.2023.102034)
Supplement: Multimedia component 1 [file mmc1.pdf]

Supplemental Table 1. Average Women's Multivitamin and Mineral (MVM) Supplement Developed from the Dietary Supplement Label Database

Common nutrients in MVM supplement intended for women 19-50y	Average amount of nutrient in Women's MVM supplements	DRI Reference: Females 19-30y (2000 kcal) 30-50y (1800 kcal)	Percent of DRI attained from Women's MVM Composite	DRI Reference: Lactation 19-30y (2400 kcal), 31-50y (2200 kcal) 0-12 months postpartum	Percent of DRI attained from Women's MVM Composite
Vitamin B_{12} (mcg)	26.64	2.4	1110.00	2.8	951.43
Vitamin B_6 (mcg)	3.73	1.3	286.92	2	186.50
Vitamin C (mg)	92.1	75	122.80	120	76.75
Calcium (mg)	277.09	1000	27.71	1000	27.71
